# Use of GeneXpert Remnants for Drug Resistance Profiling and Molecular Epidemiology of Tuberculosis in Libreville, Gabon

**DOI:** 10.1128/JCM.02257-16

**Published:** 2017-06-23

**Authors:** Amel Kévin Alame-Emane, Catherine Pierre-Audigier, Oriane Cordelia Aboumegone-Biyogo, Amandine Nzoghe-Mveang, Véronique Cadet-Daniel, Christophe Sola, Joël Fleury Djoba-Siawaya, Brigitte Gicquel, Howard E. Takiff

**Affiliations:** a Unité de Génétique Mycobacterienne, Institut Pasteur, Paris, France; b Unité de Recherche et de Diagnostics Spécialisés, Laboratoire National de Santé Publique, Libreville, Gabon; c Institute for Integrative Biology of the Cell (I2BC), CEA, CNRS, Université Paris Sud, Université Paris Saclay, Gif sur Yvette, France; d Emerging Bacterial Pathogens Laboratory, CAS Key Laboratory of Molecular Virology and Immunology, Institut Pasteur of Shanghai, Chinese Academy of Sciences, Shanghai, China; e Instituto Venezolano de Investigaciones Cientificas, Caracas, Venezuela; Carter BloodCare & Baylor University Medical Center

**Keywords:** Mycobacterium tuberculosis, Libreville, Gabon, MDR-TB, molecular epidemiology, GeneXpert, sequencing, Beijing family, drug resistance, drug sensitivity testing, multidrug resistance

## Abstract

Multidrug-resistant (MDR) and extensively drug resistant (XDR) strains of Mycobacterium tuberculosis pose major problems for global health. The GeneXpert MTB/RIF (Xpert) assay rapidly detects resistance to rifampin (RIF^r^), but for detection of the additional resistance that defines MDR-TB (MDR tuberculosis) and XDR-TB, and for molecular epidemiology, specimen cultures and a biosafe infrastructure are generally required. We sought to determine whether the remnants of sputa prepared for the Xpert assay could be used directly to find mutations associated with drug resistance and to study molecular epidemiology, thus providing precise characterization of MDR-TB cases in countries lacking biosafety level 3 (BSL3) facilities for M. tuberculosis cultures. After sputa were processed and run on the Xpert instrument, the leftovers of the samples prepared for the Xpert assay were used for PCR amplification and sequencing or for a line probe assay to detect mutations associated with resistance to additional drugs, as well as for molecular epidemiology with spoligotyping and selective mycobacterial interspersed repetitive-unit–variable-number tandem-repeat (MIRU-VNTR) typing. Of 130 sputum samples from Gabon tested with the Xpert assay, 124 yielded interpretable results; 21 (17%) of these were determined to be RIF^r^. Amplification and sequencing or a line probe assay of the Xpert remnants confirmed 18/21 samples as MDR, corresponding to 12/116 (9.5%) new and 6/8 (75%) previously treated TB patients. Spoligotyping and MIRU typing with hypervariable loci identified an MDR Beijing strain present in five samples. We conclude that the remnants of samples processed for the Xpert assay can be used in PCRs to find mutations associated with the resistance to the additional drugs that defines MDR and XDR-TB and to study molecular epidemiology without the need for culturing or a biosafe infrastructure.

## INTRODUCTION

Tuberculosis (TB) is now the leading cause of death from an infectious disease worldwide, with an estimated 10.4 million new cases and 1.4 million deaths in 2015 (1). Of these, an estimated 480,000 new cases were multidrug-resistant TB (MDR-TB), caused by strains resistant to at least isoniazid (INH) and rifampin (RIF). There are effective multidrug regimens that can cure 80% of MDR-TB cases in 9 to 12 months (2–4), but the MDR-TB cases must first be detected (5). When an MDR strain is also resistant to the fluoroquinolones (FQ) and an injectable agent, the disease is termed extensively drug resistant TB (XDR-TB) and is unlikely to be cured with standard MDR treatment regimens (6).

Africa accounts for about one-quarter of the world's TB cases, but few countries in the region have detailed data on TB management and the prevalence of MDR cases (7). Gabon is a country in Central Africa with about 1.7 million inhabitants, half of whom live in the capital, Libreville. The World Health Organization (WHO) estimated that in 2014, Gabon had an annual incidence of 393 to 497 cases, with an accompanying mortality rate of 56 to 93 TB deaths per 100,000 inhabitants, making it one of the 10 highest-incidence countries (7). Between 8 and 11% of Gabonese TB patients are suspected of having MDR-TB (9), and a recent report from Lambaréné, Gabon, found that 42% of adult and 16% of pediatric TB patients were coinfected with the human immunodeficiency virus (HIV) (10).

The high incidence of TB cases and TB mortality reflects the deficiencies in Gabon's TB control program (11–13). A survey in 2006 found that only 55% of patients, whose strains were not routinely tested for drug resistance, completed the standard first-line drug regimen, and a more recent study found only 53% treatment success (7, 10–12). There is no public health laboratory in Gabon with the ability to perform phenotypic drug susceptibility testing (DST) (5), and MDR drug regimens are not administered with standardized protocols in most of the country. However, a WHO-approved MDR treatment program is currently being implemented, and therefore, identification of MDR-TB patients to receive this treatment has become a priority. The GeneXpert MTB**/**RIF assay (Xpert assay; Cepheid, Sunnyvale, CA, USA), was recently introduced into the country and can rapidly analyze sputum samples to detect the presence of Mycobacterium tuberculosis bacilli, as well as mutations associated with resistance to rifampin (RIF^r^), a marker for MDR-TB (14, 15). The Xpert assay does not, however, detect the mutations associated with the additional drug resistance that defines XDR-TB.

We report here a novel method, not requiring cultures or a biosafe infrastructure, that can detect resistance to drugs other than rifampin. The method first employs the Xpert assay to detect the presence of M. tuberculosis bacilli and RIF^r^; it then uses the remainder of the sputum samples prepared for the Xpert assay in PCRs to identify mutations associated with resistance to additional drugs as well as to define the lineage of circulating TB strains. This strategy was used to profile the nature and extent of drug resistance in Libreville, Gabon.

## RESULTS

### Use of Xpert remnants for amplification and sequencing to find mutations conferring resistance to first- and second-line drugs.

**FIG 1:**
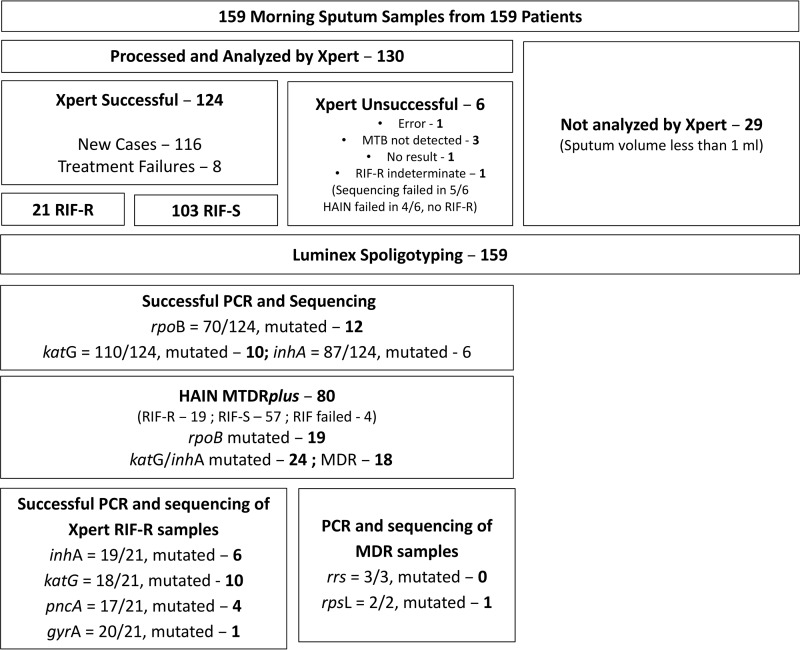
Flow chart showing the processing of the 159 sputum samples.

Of the 130 samples run on the Xpert instrument, 1 gave a no-result signal, 1 gave an error signal, 1 was positive for M. tuberculosis but indeterminate for RIF^r^, and M. tuberculosis was not detected in 3. Among the remaining 124 Xpert samples, RIF^r^ was detected in 21 (17%), of which 12 were mutated at RpoB amino acid residue 531 or 533, and 9 were mutated at RpoB residue 516 or 522 (Table 1; Fig. 1).

**TABLE 1:** Mutations determined by the GeneXpert MTB/RIF assay, the Hain MTBDR*plus* test, and sequencing of the individual genes, performed with leftovers of the Xpert processed sputum specimens

Sample	Patient^*a*^		GeneXpert result		*rpoB* result		MDR	*katG* result (codon 315)		*inhA* promoter result (position −15)		Sequencing result for:			
	Sex	Age (yr)	Probe(s)^*b*^	Mutation(s) detected at codon(s):	Sequencing^*c*^	Hain test		Sequencing	Hain test	Sequencing	Hain test	*pncA*	*rrs*	*rpsL*	*gyrA*
GAB-001	M	54	B and C	516 or 522	D516Y	Mutated (probes WT4 and -5 absent)	x	S315T	S315T	WT	WT	WT			WT
GAB-065	F	19	B and C	516 or 522	D516Y	Mutated (probe WT4 absent)	x	S315T	S315T	WT	WT	WT			WT
GAB-152	F	18	B	516	D516Y	Mutated (probe WT4 absent)	x	S315T	S315T	WT	WT	WT			WT
GAB-076	F	26	C	522	D516Y, S522T	Mutated (probe WT4 absent)	x	S315T	S315T	WT	WT	WT			WT
GAB-163	F	23	E	531 or 533	NS	S531L	x	S315T	S315T	WT	WT	NS			WT
GAB-003	M	35	E	531 or 533	S531L	S531L	x	S315T	S315T	WT	WT	WT			WT
GAB-009	F	37	E	531 or 533	S531L	S531L	x	S315T	S315T	WT	WT	G97S			WT
GAB-182	M	32	E	531 or 533	S531L	S531L	x	S315T	S315T	WT	WT	T135P			WT
GAB-002	F	50	B	516	D516Y	Mutated (probe WT4 absent)	x	S315T	S315T	WT	C → T	WT	WT	K43R	D94G
GAB-180	M	19	B	516	D516Y	Mutated (probe WT4 absent)	x	S315T	S315T	WT	C → T	WT			WT
GAB-014	M	30	E	531 or 533	NS	Mutated (probe WT8 absent)	x	WT	WT	C → T	C → T	NS	WT		NS
GAB-173	M	44	E	531 or 533	S531L	S531L	x	WT	WT	C → T	C → T	WT	WT	WT	WT
GAB-062	M	18	E	531 or 533	NS	S531L	x	WT	WT	C → T	C → T	WT			WT
GAB-151	F	42	E	531 or 533	NS	S531L	x	WT	WT	C → T	C → T	WT			WT
GAB-157	M	25	E	531 or 533	NS	S531L	x	WT	WT	C → T	C → T	WT			WT
GAB-010	F	28	E	531 or 533	S531L	S531L	x	WT	WT	C → T	WT	S88Stop			WT
GAB-068	F	54	E	531 or 533	NS	S531L	x	NS	S315T	WT	WT	G97S			WT
GAB-072	M	23	C	522	NS	Mutated (probes WT3 and -4 absent)	x	NS	S315T	NS	WT	WT			WT
GAB-017	M	26	E	531 or 533	S531L	S531L		WT	S315T	WT	WT	NS			WT
GAB-059	F	32	B	516	NS	WT		NS	WT/S315T^*d*^	WT	WT	WT			WT
GAB-191	?	?	B	516	NS	TUB^*e*^ control absent		WT	WT	NS	C → T	NS			WT

a M, male; F, female; ?, unknown.

b Xpert probe B can also detect less-frequent mutations affecting amino acid 513 and a deletion of amino acids 516 and 517 (31).

c NS, PCR amplification was not successful.

d Mixed profile; see Results.

e TUB, M. tuberculosis complex specific.

We then wanted to determine whether the leftover sputa processed in the Xpert assay could be used with other tests to obtain information on additional drug resistance. The Hain GenoType MTBDR*plus* assay is a line probe assay that detects rifampin resistance-associated mutations in *rpoB*, as well as mutations in *katG* (16) and *inhA* (17) that are associated with resistance to isoniazid (INH). The Hain assay was performed using Xpert-processed leftovers in order to confirm the Xpert *rpoB* results and to identify strains that were also INH resistant, thus confirming them as MDR-TB (Table 1). The Hain test found *rpoB* mutations in 19/21 samples that were RIF^r^ by the Xpert assay and also detected the KatG S315T substitution in 14/21 samples and the C → T mutation at position −15 in the *inhA* promoter in 8 samples, with GAB-180 and GAB-002 showing both *katG* and *inhA* mutations. In one of the two *rpoB*-discordant samples (GAB-059), where the Hain test did not confirm the presence of an *rpoB* mutation found by the Xpert assay, the Hain test detected a mixed KatG profile with positive results for both the wild-type (WT) sequence and the S315T substitution, suggesting that the sample might have contained a mixed population of two strains of M. tuberculosis. In that case, the *rpoB* codon 516 mutation detected by the Xpert assay could have been hidden in the Hain test by the presence of a WT *rpoB* sequence. In the other discordant strain (GAB-191), the RIF resistance mutation detected by the Xpert assay was not found with the Hain test, but because there was no hybridization to the M. tuberculosis complex-specific (TUB) control band, the Hain test for this sample was not valid.

To confirm which strains were MDR, the Xpert leftovers were used in PCRs to amplify the *rpoB*, *katG*, and *inhA* genes for DNA sequencing (Fig. 1). Readable sequences were obtained from 70/124 samples (56%) for *rpoB*, of which 12 had mutations; from 110/124 samples (89%) for *katG*, of which 10 had mutations; and from 87/124 (67%) samples for *inhA*, of which 6 had mutations. The 12 mutated *rpoB* sequences contained the same mutations found with the Xpert and Hain tests (Table 1), except that for sample GAB-076, the Xpert assay detected the codon 522 mutation while the Hain test detected the codon 516 mutation, but sequencing showed that both mutations were present. Unfortunately, sequencing of the *rpoB* gene was not successful for either of the two samples where the Hain test did not confirm the Xpert results (GAB-059 and GAB-191). Among the 21 samples found to be RIF^r^ by the Xpert assay, readable *katG* sequences were obtained from 18, of which 10 had mutations resulting in the S315T substitution, and readable *inhA* sequences were obtained from 19, of which 6 carried the C → T mutation at position −15 in the promoter. Despite repeated attempts, it was not possible to amplify the *rpoB* gene from 9 samples, or the *katG* or *inhA* gene from 3 or 2 samples, respectively.

Most of the sequences yielded the same results found by the Hain test, but a few were discordant. For two samples (GAB-002 and -180), the Hain test detected the C → T mutation at position −15 in the *inhA* promoter, which was not present in the sequences, but *katG* mutations were detected in these samples by both the Hain test and sequencing, making them MDR. Sample GAB-010 was WT for both *katG* and *inhA* by the Hain test but showed the C → T mutation at position −15 in the *inhA* promoter by sequencing and was therefore classified as MDR.

Based on sequencing results or a Hain test without a discordant sequence, 18 of the 21 samples (86%) determined to be RIF^r^ by the Xpert assay would be classified as MDR. Results for the remaining three samples were as follows. For GAB-017, the Hain test found a KatG S315T substitution, but the *katG* sequence was WT. For GAB-191, the Hain test found the *inhA* promoter mutation, but the absence of a positive control invalidated the Hain test, and no *inhA* sequence was available. For GAB-059, the Hain test did not detect an *rpoB* mutation and showed both mutated and WT *katG* sequences, suggesting the possible presence of two strains, one WT and one MDR with *rpoB* and *katG* mutations.

To detect additional antibiotic resistance in the 21 samples determined to be RIF^r^ by the Xpert assay, we amplified and sequenced the *pncA*, *gyrA*, *rrs*, and *rpsL* genes for detection of mutations associated with resistance to pyrazinamide (PZA), fluoroquinolones, the injectable antibiotics, and streptomycin, respectively. Readable sequences were obtained for *pncA* in 17/21 (81%) samples, of which 4 (24%) carried mutations (Table 1; Fig. 1). For the *gyrA* gene, 20/21 (95%) samples yielded readable sequences, of which 1 (5%) was mutated. The *rrs* gene was amplified and sequenced from the only three samples that could contain XDR-TB: the sample with the *gyrA* mutation, the sample for which sequencing of *gyrA* was unsuccessful, and a sample from a patient who had received 7 months of treatment with streptomycin (SM). All three were WT for the *rrs* gene, showing that there were no XDR-TB cases. The *rpsL* gene was sequenced from the samples of two patients: the patient treated with SM (GAB-173) had a WT *rpsL* sequence; but the sample with the *gyrA* mutation (GAB-002) also had a mutation causing a K43R substitution in RpsL, making this MDR-FQ^r^ sample also SM^r^.

### Epidemiology of MDR-TB patients.

Epidemiological data for MDR-TB patients are shown in Table 2. Of the 18 patients confirmed to have MDR-TB strains, 9 were male and 9 were female. The mean age was 32 years (range, 18 to 54), and 2 patients were non-Gabonese. Of the 8 patients who had been treated with first-line drugs previously, 6 (75%) had samples that were MDR. One of these 6 patients also had mutations associated with resistance to FQ and SM, and 2 others had mutations associated with resistance to PZA. Of the 116 individuals with new TB cases, 12 (10.3%) had strains that were MDR. Of these 12, 6 (50%) had not started TB treatment when the samples were taken. One of their strains had a *pncA* mutation. The remaining 6 individuals with new MDR-TB cases had begun first-line treatment 2, 3, 4 (2 patients), 6, or 8 months prior to the time the samples were obtained.

**TABLE 2:** Epidemiology and spoligotypes of sputum samples determined by the Xpert assay to be RIF^r^

Sample	Patient		Spoligotype	Additional resistance^*a*^	SIT	Clade	Medical information		Demographic information		Laboratory and Xpert results	
	Sex	Age (yr)					Exam^*b*^	Status^*c*^	District	Nationality	Microscopy score^*d*^	Xpert score
GAB-157	M	25	◼◼◼◼◼◼◼◼◼◼◼◼◼◼◼◼◼◼◼◼◼◼◻◻◼◼◼◼◼◼◼◼◻◻◼◼◼◼◼◼◼◼◼	INH	Orphan		C4	N	Akanda	Gabon	1+	Medium
GAB-065	F	19	◻◻◻◻◻◻◻◻◻◻◻◻◻◻◻◻◻◻◻◻◻◻◻◻◻◻◻◻◻◻◻◻◻◻◼◼◼◼◼◼◼◼◼	INH	1	Beijing	D	N	Owendo	Gabon	2+	Medium
GAB-001	M	54	◻◻◻◻◻◻◻◻◻◻◻◻◻◻◻◻◻◻◻◻◻◻◻◻◻◻◻◻◻◻◻◻◻◻◼◼◼◼◼◼◼◼◼	INH	1	Beijing	C	FR	ND	Gabon	10 AFB	Medium
GAB-072	M	23	◻◻◻◻◻◻◻◻◻◻◻◻◻◻◻◻◻◻◻◻◻◻◻◻◻◻◻◻◻◻◻◻◻◻◼◼◼◼◼◼◼◼◼	INH	1	Beijing	C6	N	5th	Gabon	1+	Very low
GAB-076	F	26	◻◻◻◻◻◻◻◻◻◻◻◻◻◻◻◻◻◻◻◻◻◻◻◻◻◻◻◻◻◻◻◻◻◻◼◼◼◼◼◼◼◼◼	INH	1	Beijing	C4	N	Akanda	Gabon	2+	Medium
GAB-180	M	19	◻◻◻◻◻◻◻◻◻◻◻◻◻◻◻◻◻◻◻◻◻◻◻◻◻◻◻◻◻◻◻◻◻◻◼◼◼◼◼◼◼◼◼	INH	1	Beijing	C2	N	5th	Gabon	1+	High
GAB-003	M	35	◼◼◼◼◼◼◼◼◼◼◼◼◼◼◼◼◼◼◼◼◼◼◼◼◼◼◼◼◼◼◻◼◻◻◻◻◼◼◼◼◼◼◼	INH	50	H3	D	FR	5th	Gabon	3+	Medium
GAB-163	F	23	◼◼◼◼◼◼◼◼◼◼◼◼◼◼◼◼◼◼◼◼◼◼◼◼◼◼◼◼◼◼◻◼◻◻◻◻◼◼◼◼◼◼◼	INH	50	H3	D	N	6th	Gabon	2+	High
GAB-182	M	32	◼◼◼◼◼◼◼◼◼◼◼◼◼◼◼◼◼◼◼◼◼◼◼◼◼◼◼◼◼◼◻◼◻◻◻◻◼◼◼◼◼◼◼	INH, PZA	50	H3	C3	FR	2nd	Other	1+	Medium
GAB-014	M	30	◼◼◼◼◼◼◼◼◼◼◼◼◼◼◼◼◼◼◼◼◼◼◼◼◼◼◼◼◼◼◼◼◻◻◻◻◼◼◼◼◼◼◼	INH	53	T1	D	N	5th	Gabon	1+	Medium
GAB-002	F	50	◻◻◻◻◻◻◻◻◻◻◻◻◻◻◻◻◻◻◻◻◻◻◻◻◻◻◻◻◻◻◻◻◻◻◼◼◻◻◼◼◼◼◼	INH, FQ, SM	260	Beijing	C	FR	5th	Gabon	3+	High
GAB-151	F	42	◼◼◼◼◼◼◼◼◼◼◼◼◼◼◼◼◼◼◼◼◼◼◻◻◼◼◼◼◼◼◼◼◻◻◻◻◼◻◻◼◼◼◼	INH	370	T1	D	N	2nd	Gabon	1+	Medium
GAB-173	M	44	◼◼◼◼◼◼◼◼◼◼◼◼◼◼◼◼◼◼◼◼◼◼◻◻◼◼◼◼◼◼◼◼◻◻◻◻◼◻◻◼◼◼◼	INH	370	T1	C5	FR	2nd	Other	7 AFB	High
GAB-068	F	54	◼◼◼◼◼◼◼◼◼◼◼◼◼◼◼◼◼◼◼◼◼◼◼◼◼◼◼◼◼◼◼◼◻◻◻◼◼◼◼◼◼◼◼	INH, PZA	1196		C3	N	1st	Gabon	1+	Medium
GAB-009	F	37	◼◼◼◼◼◼◼◼◼◼◼◼◼◼◼◼◼◼◼◼◼◼◼◼◼◼◼◼◼◼◼◼◻◻◻◼◼◼◼◼◼◼◼	INH, PZA	1196		D	FR	Owendo	Gabon	2+	High
GAB-010	F	28	◼◼◼◼◼◼◼◼◼◼◼◼◼◼◼◼◼◼◼◼◼◼◼◼◼◼◼◼◼◼◼◼◻◻◻◼◼◼◼◼◼◼◼	INH, PZA	1196		D	N	6th	Gabon	2+	Medium
GAB-062	M	18	◼◼◼◼◼◼◼◼◼◼◼◼◼◼◼◼◼◼◼◼◼◼◻◻◼◼◼◼◼◼◼◼◻◻◻◻◼◼◼◼◼◼◼	INH	1580	T1	D	N	2nd	Gabon	1+	Medium
GAB-017	M	26	◼◼◼◼◼◼◼◼◼◼◼◼◼◼◼◼◼◼◼◼◼◼◻◻◻◼◼◼◼◼◼◼◻◻◼◼◼◼◼◼◼◼◼	INH^*e*^	2697	MANU2	D	FR	2nd	Gabon	2+	Very low
GAB-152	F	18	◼◼◼◼◼◼◼◼◼◼◼◼◼◼◼◼◼◼◼◼◼◼◻◻◻◼◼◼◼◼◼◼◻◻◼◼◼◼◼◼◼◼◼	INH	2697	MANU2	C8	N	1st	Gabon	7 AFB	Medium
GAB-059	F	32	◼◼◼◼◼◼◼◼◼◼◼◼◼◼◼◼◼◼◼◼◼◼◻◻◻◼◼◼◼◼◼◼◻◻◼◼◼◼◼◼◼◼◼	INH^*f*^	2697	MANU2	D	N	5th	Gabon	1+	Low
GAB-191	ND	ND	◻◻◻◻◻◼◻◻◻◻◻◻◻◻◻◻◻◻◻◻◻◻◻◻◻◻◻◻◼◻◻◻◼◻◼◼◼◼◼◼◼◼◼		Orphan		ND	ND	ND	Gabon	ND	Medium

a INH, isoniazid; PZA, pyrazinamide; FQ, fluoroquinolones; SM, streptomycin.

b C, control microscopy (with the number of months for which the individual had been under treatment with first-line drugs given in parentheses); D, initial microscopy for diagnosis; ND, no data.

c N, new TB case; FR, failed or relapsed case.

d 3+, >10 acid-fast bacilli (AFB) per high-power field (HPF); 2+, 1 to 10 AFB per HPF; 1+, 10 to 99 AFB per 100 HPFs. If there are <10 AFB per 100 HPFs, the absolute number of AFB observed in 100 HPFs is shown.

e For this strain, the Hain test found an S315T KatG substitution, but sequencing found WT *katG*.

f This strain was RIF^r^ by the Xpert assay but had a WT *rpoR* sequence by the Hain test, which also detected both WT *katG* and *katG* S315T. The Hain test detected a C → T mutation at position −15 in the *inhA* promoter, while sequencing found WT *inhA*.

### Molecular epidemiology of MDR strains.

Spoligotypes were obtained for all 124 samples tested by the Xpert assay, as well as for the 29 samples with volume insufficient for the Xpert assay and the 6 samples for which Xpert analysis was unsuccessful (Fig. 1). The spoligotypes of two strains grouped with Mycobacterium africanum West African 1. The most frequent spoligotyping international type (SIT) was SIT61, found in 42 isolates (Table 3), but none of these were RIF^r^ by any method used. In contrast, RIF^r^ was found in 5 of 5 ST1, 3 of 16 SIT50, 3 of 6 SIT1196, 2 of 6 SIT370, 3 of 6 SIT2697, 1 of 2 SIT1580, 1 of 6 SIT53, and 1 of the 17 orphan isolates (Table 2). The single sample with SIT260, which, like ST1, belongs to the Beijing family, was also MDR and, additionally, FQ^r^ and SM^r^.

**TABLE 3:** Clustered spoligotypes^*a*^

SIT	Clade	No. of isolates (% of total)	Spoligotype	Octal
61	LAM10-CAM	42 (26.42)	◼◼◼◼◼◼◼◼◼◼◼◼◼◼◼◼◼◼◼◼◼◼◻◻◻◼◼◼◼◼◼◼◻◻◻◻◼◼◼◼◼◼◼	777777743760771
50	H3	16 (10.1)	◼◼◼◼◼◼◼◼◼◼◼◼◼◼◼◼◼◼◼◼◼◼◼◼◼◼◼◼◼◼◻◼◻◻◻◻◼◼◼◼◼◼◼	777777777720771
53	T1	6 (3.8)	◼◼◼◼◼◼◼◼◼◼◼◼◼◼◼◼◼◼◼◼◼◼◼◼◼◼◼◼◼◼◼◼◻◻◻◻◼◼◼◼◼◼◼	777777777760771
54	MANU2	6 (3.8)	◼◼◼◼◼◼◼◼◼◼◼◼◼◼◼◼◼◼◼◼◼◼◼◼◼◼◼◼◼◼◼◼◻◻◼◼◼◼◼◼◼◼◼	777777777763771
370	T1	6 (3.8)	◼◼◼◼◼◼◼◼◼◼◼◼◼◼◼◼◼◼◼◼◼◼◻◻◼◼◼◼◼◼◼◼◻◻◻◻◼◻◻◼◼◼◼	777777747760471
1196		6 (3.8)	◼◼◼◼◼◼◼◼◼◼◼◼◼◼◼◼◼◼◼◼◼◼◼◼◼◼◼◼◼◼◼◼◻◻◻◼◼◼◼◼◼◼◼	777777777761771
2697	MANU2	6 (3.8)	◼◼◼◼◼◼◼◼◼◼◼◼◼◼◼◼◼◼◼◼◼◼◻◻◻◼◼◼◼◼◼◼◻◻◼◼◼◼◼◼◼◼◼	777777743763771
1	Beijing	5 (3.1)	◻◻◻◻◻◻◻◻◻◻◻◻◻◻◻◻◻◻◻◻◻◻◻◻◻◻◻◻◻◻◻◻◻◻◼◼◼◼◼◼◼◼◼	000000000003771
42	LAM9	4 (2.5)	◼◼◼◼◼◼◼◼◼◼◼◼◼◼◼◼◼◼◼◼◻◻◻◻◼◼◼◼◼◼◼◼◻◻◻◻◼◼◼◼◼◼◼	777777607760771
523	MANU_ancestor	4 (2.5)	◼◼◼◼◼◼◼◼◼◼◼◼◼◼◼◼◼◼◼◼◼◼◼◼◼◼◼◼◼◼◼◼◼◼◼◼◼◼◼◼◼◼◼	777777777777771
237		3 (1.9)	◼◼◼◼◼◼◼◼◼◼◼◼◼◼◼◼◼◼◼◼◼◼◼◼◼◼◼◼◼◼◻◻◻◻◻◻◻◻◻◻◻◻◻	777777777700000
378	T1	3 (1.9)	◼◼◼◼◼◼◼◼◼◼◼◼◼◼◼◼◼◼◼◼◻◼◼◻◼◼◼◼◼◼◼◼◻◻◻◻◼◼◼◼◼◼◼	777777667760771
2298	T1	3 (1.9)	◼◼◼◼◼◼◼◼◼◼◼◼◼◼◼◼◼◼◼◼◻◼◻◻◼◼◼◼◼◼◼◼◻◻◻◻◼◼◼◼◼◼◼	777777647760771
176	LAM6	2 (1.3)	◼◼◼◼◼◼◼◼◼◼◼◼◻◼◼◼◼◼◼◼◻◻◻◻◼◼◼◼◻◼◼◼◻◻◻◻◼◼◼◼◼◼◼	777737607560771
373	T1	2 (1.3)	◼◼◼◼◼◼◼◼◼◼◼◼◼◼◼◼◼◼◼◼◼◼◼◻◼◼◼◼◼◼◼◼◻◻◻◻◼◼◼◼◼◼◼	777777767760771
741	H3	2 (1.3)	◼◼◼◼◼◼◼◼◼◼◼◼◼◼◼◼◼◼◼◼◼◼◻◼◼◼◼◼◼◼◻◼◻◻◻◻◼◼◼◼◼◼◼	777777757720771
1580	T1	2 (1.3)	◼◼◼◼◼◼◼◼◼◼◼◼◼◼◼◼◼◼◼◼◼◼◻◻◼◼◼◼◼◼◼◼◻◻◻◻◼◼◼◼◼◼◼	777777747760771
1690	MANU2	2 (1.3)	◼◼◼◼◼◼◼◼◼◼◼◼◼◼◼◼◼◼◼◼◼◼◼◼◼◼◼◼◼◼◼◼◻◼◻◻◼◼◼◼◼◼◼	777777777762771

a Of 159 total isolates, 120 (75.5%) belonged to clustered spoligotypes; 22 (13.8%) had unique SITs, and 17 (10.7%) were orphan strains.

The standard mycobacterial interspersed repetitive-unit–variable-number tandem-repeat (MIRU-VNTR) loci are poorly discriminatory for Beijing strains, so in order to determine whether the five SIT1 Beijing MDR strains belonged to the same genotype, they were analyzed with 4 VNTR systems (VNTR systems 1982, 3232, 3820, and 4120) reported to be hypervariable for Beijing strains (18, 19). VNTR systems 1982, 3820, and 4120 yielded identical bands for the five SIT1 Beijing MDR samples (see Fig. S1 in the supplemental material), suggesting that they all belonged to the same genotype. Amplification with primers for system 3232 yielded several bands per sample and was considered uninterpretable. Sequencing of the Rv2629 gene showed that all five strains had the 191C genotype, described as a marker for the Beijing-W clade, cluster group 2 (20).

## DISCUSSION

The Xpert technology has improved TB diagnosis and can rapidly detect RIF^r^, which, as confirmed in this study, is a reliable indicator of MDR-TB (21). However, most methods for detecting resistance to antibiotics other than rifampin require culturing of the specimen. Cultures on solid media take more than 2 weeks to turn positive, while cultures in liquid media can turn positive in 7 to 10 days (22) but pose a greater biosafety hazard. The Hain MTBDR*plus* test can be performed directly on sputum specimens but detects resistance to RIF and INH only. Amplification and sequencing of Xpert leftovers, the alternative method proposed here, yielded readable *inhA* sequences for 19, and readable *katG* sequences for 18, of the 21 strains determined to be RIF^r^ by the Xpert assay. This method confirmed that 18/21 strains were unequivocally MDR. In four instances, the sequence was discordant with the Hain test result (GAB-002, -0180, -010, and -017), but two of these samples (GAB-002 and 180) were nonetheless defined as MDR because both sequencing and the Hain test detected substitutions at KatG residue 315. The other two had confirmed *rpoB* mutations and a mutation associated with INH resistance detected by only one technique, but for the sake of caution, these should probably be regarded as MDR despite the discordant results. The *rpoB* discrepancy for GAB-059, together with the presence of signals for both WT and mutated *katG*, suggests a mixed infection with both sensitive and resistant bacilli.

Testing for FQ resistance in MDR strains is critical, because treatment failures with FQ-containing MDR regimens are more likely when the strain is FQ^r^, although MDR strains with particular *gyrA* mutations may be cured with higher FQ doses (23). The Hain MTBDR*sl* test can detect resistance to FQ and injectable antibiotics, and a new version, which appeared after the present study was completed, can be used directly with sputum specimens (24). It might be worthwhile to compare the performance of this test on Xpert leftovers with its results when it is used directly on sputa.

Our alternative method for detecting XDR-TB, using the leftovers from sputa processed for the Xpert assay to amplify and sequence the *gyrA* and *rrs* genes, is easy, quick, and relatively inexpensive. The sequencing can be done by a commercial service, and if the specimens are processed rapidly and promptly sent to be sequenced, the results could be available by Internet within about 7 days after the sputum specimen is obtained. Although amplification of the *rpoB* gene was successful for only 12 of 21 samples, it is unlikely that this low success rate was due to inhibitors in the stored Xpert leftovers, because readable sequences were obtained from 18/21 samples for *katG*, 19/21 samples for *inhA*, and 20/21 samples with *gyrA*. Perhaps the success rate with *rpoB* could be improved by optimizing the primers. The success rates of sequencing and the Hain test were roughly the same for sputa with 1+, 2+, or 3+ positivity but were lower for sputa with fewer than 1+ bacilli on microscopy and would probably be lower still for smear-negative specimens in which the Xpert assay detects M. tuberculosis and RIF^r^.

Xpert leftovers were also used for molecular epidemiology studies with both spoligotyping and MIRU-VNTR typing. Spoligotyping with the Luminex format showed that most of the MDR strains belonged to spoligotypes that were also present in non-MDR strains, but surprisingly, there were no MDR isolates with the most frequent spoligotype, SIT61, which was seen in 26% of all isolates examined (42/159). An alarming finding was that 6 of the 18 confirmed MDR strains belonged to SIT1 or SIT260, genotypes of the Beijing family that has been associated with many MDR-TB outbreaks (25, 26). Because MIRU-VNTR typing, even with 24 loci, lacks discriminatory power for the Beijing family, we used VNTR loci reported to be hypervariable in the Beijing lineage, which confirmed that the five SIT1 strains apparently belonged to the same genotype (18, 19). SIT1 was found only in these five MDR strains, and SIT260 was found only in one strain that was MDR, FQ^r^, and SM^r^. This suggests that while most of the MDR strains could have developed drug resistance within Gabon, the SIT1 strains and the SIT260 strain may have been MDR when they were introduced into Libreville. The origin of these strains is a mystery, since reports from neighboring countries suggest that Beijing strains are not common in the region, and those found were generally not MDR (27–29). Follow-up studies are in progress to determine the extent and epidemiology of the apparent SIT1 MDR outbreak strain and to see if the MDR, FQ^r^, SM^r^ SIT260 Beijing strain is also spreading.

The strategy employed in this study provided important information on the urgent tuberculosis problem in Gabon, without the need for cultures or biohazard facilities. Because only 124 strains were examined in this study, and the sample was not representative of the entire country, the exact percentages of MDR-TB we found, 10% of new cases and 75% (6/8) of previously treated cases, may not accurately represent the situation for all of Gabon, but these high rates are nevertheless ominous and are likely attributable to the low success rates (only 53 to 55%) reported for first-line therapy in Gabon (10).

This method could be used to efficiently provide information on drug resistance and molecular epidemiology in other low-resource countries and can likely be used with any molecular test based on the amplification of specific genes or loci (30). Methods for phenotypic drug sensitivity testing rely on cultures and require a biosafe infrastructure to reduce the biohazards inherent in the manipulation of clinical samples, and heat inactivation of bacteria can generate aerosol risks. In contrast, the bacilli in samples processed for the Xpert assay are no longer viable (31), so they can be used in PCRs without any special infrastructure.

Whole-genome sequencing (WGS) has been proposed as a more-comprehensive and safer alternative to phenotypic drug testing and molecular epidemiology (32, 33). With advances in technology and cost reductions, WGS performed directly on clinical samples suspected to be MDR or XDR could eventually be cost-effective, even in low-resource settings. In a possible future algorithm, Xpert leftovers of RIF^r^ samples could be sent for WGS to determine the full resistance profile, but this would require better sequencing technology and improved methods for isolating M. tuberculosis DNA from clinical specimens (34, 35). The strategy used in this study could represent an efficient interim solution for finding mutations associated with resistance to second- and third-line drugs, especially in settings where phenotypic drug sensitivity testing is not possible, and also for basic or retrospective molecular epidemiology.

## MATERIALS AND METHODS

### Specimens.

Between October 2014 and February 2015, 159 morning sputum samples were collected from 159 microscopy-positive patients who either were newly diagnosed with TB or were not cured after two courses of standard first-line therapy. Some patients had also received streptomycin (SM). The patients presented to one of the three main TB diagnostic laboratories in Libreville, the capital of Gabon: Nkembo Respiratory Hospital (124 samples), the National Public Health Laboratory (LNSP) (31 samples), and the Melen regional hospital (4 samples). The bacilli were seen in all samples after Ziehl-Neelsen staining and were scored as +/−, 1+, 2+, or 3+ (36).

The Ethics Committee of the Gabon LNSP approved the study (Décision 23022015-1). It was undertaken in the context of a research agreement between the Institut Pasteur of Paris, France, and the Government of Gabon. After a written or oral explanation, patients signed an informed consent to allow the use of their sputa in the study and to provide sociodemographic data and information on previous TB treatment. All sputum samples were transported to the National Public Health Laboratory and were processed for the Xpert assay.

Before the addition of the Xpert diluent, 29 sputum samples, 28 from new cases and 1 from a previously treated patient, had less than the 1-ml volume considered to be the minimum required for the Xpert assay (15). These samples were processed and, though not assayed with the Xpert test (Fig. 1), were used in PCRs for spoligotyping. After the specified volumes of the 130 processed sputum samples were run on the Xpert system, 375 μl of each of the leftover diluted samples was individually placed in a 50-ml tube containing 25 ml of autoclaved phosphate buffer (Na_2_HPO_4_·2H_2_O and KH_2_PO_4_ at 0.067 M [pH 6.8]) to neutralize the alkaline pH of the Xpert leftover to pH 7.0. After centrifugation at 4,500 rpm for 15 min, the pellets were resuspended in 100 μl 1× TE (10 mM Tris-HCl, 1 mM disodium EDTA [pH 8]) and were transferred to a microtube. The microtube was heated at 90°C for 30 min in a water bath and was frozen at −40°C for 1 h. After thawing, the lysate was centrifuged at 8,000 rpm for 5 min, and the supernatant was transferred to a new microtube and was stored at −40°C until it was sent to the Unité de Génétique Mycobacterienne at the Institut Pasteur in Paris, France, where the rest of the studies were performed. The 29 samples not run on the Xpert system were similarly processed.

### Detection of drug resistance mutations.

The processing of the samples is shown in Fig. 1. PCR was performed with primers targeting *rpoB*, *katG*, *inhA*, *pncA*, *gyrA*, *rrs*, and *rpsL* (Table 4) to detect mutations conferring resistance to RIF, isoniazid (INH), pyrazinamide (PZA), the fluoroquinolones (FQ), amikacin (AMK), kanamycin (KAN), capreomycin (CPN), and SM (Table 4) (37–39). The *rpoB*, *katG*, *inhA*, *pncA*, *gyrA*, and *rpsL* genes were amplified separately in 50-μl reaction mixtures containing 1× NH_4_ buffer (Bioline), 10% dimethyl sulfoxide (DMSO), 1.5 mM MgCl_2_, 0.3 μM each primer, 200 μM deoxynucleoside triphosphates (dNTP), 1 U BioTaq DNA polymerase (Bioline), and 5 μl of the Xpert sample remnant diluted 1:10 in water. The thermocycler program was as follows: 95°C for 5 min; 45 cycles of 95°C for 1 min, the hybridization temperature (Table 4) for 1 min, and 72°C for 1 min; and a final extension at 72°C for 7 min. The *rrs* gene was amplified in a 30-μl reaction mixture containing 1× GC buffer (TaKaRa), 400 μM dNTP, 0.4 μM each primer, 1 U TaKaRa LA *Taq* (TaKaRa Bio Inc., Japan) and 5 μl of the Xpert sample remnant diluted 1:10 in water. The amplification program was as follows: 94°C for 5 min; 35 cycles of 94°C for 30 s, 59°C for 30 s, and 72°C for 2 min; and a final extension at 72°C for 5 min. Ten microliters of each PCR product was electrophoresed on a 1% agarose gel to verify the amplification. The PCR products were sequenced with the forward and reverse amplification primers (Cochin Sequencing Platform, Paris, France), and the sequencing results were aligned to the respective wild-type (WT) genes of M. tuberculosis H37Rv using Geneious (v9.04), MEGA (v6.06), or BLAST (www.ncbi.nlm.nih.gov). For all strains without readable *rpoB* sequences and all strains that were RIF^r^ by the Xpert assay, the Hain GenoType MTBDR*plus* assay, v2.0 (Hain Lifescience, Nehren, Germany), was used to detect mutations in *rpoB* (31), *katG*, and *inhA* (40) according to the manufacturer's protocol, using 5 μl of the undiluted Xpert sample remnant.

**TABLE 4:** Primers used to amplify and sequence the Mycobacterium tuberculosis genes associated with resistance to antibiotics

Gene	Antibiotic	Primer name	Primer sequence (5′–3′)	Hybridization temp (°C)	Product size (bp)
*rpoB*	Rifampin	TR1	TACGGTCGGCGAGCTGATCC	53	411
		TR2	TACGG CGTTTCGATGAACC		
*katG*	Isoniazid	katG1	TGGCCGCGGCGGTCGACATT	60	330
		katG2	CCAGCAGGGCTCTTCGTCAG		
*inhA*	Isoniazid	inhA promo-1	CCTCGCTGCCCAGAAAGGGA	60	248
		inhA promo-2	ATCCCCCGGTTTCCTCCGGT		
*pncA*	Pyrazinamide	*pnc*A1	ATCGCGATGGAACGTGATA	60	950
		*pnc*A2	CTGTCACCGGACGGATTTG		
*gyrA*	Fluoroquinolones	*gyr*A-F	GATGACAGACACGACGTTGC	55	398
		*gyr*A-R	GGGCTTCGGTGTACCTCAT		
*gyrB*	Fluoroquinolones	*gyr*B-F	CCACCGACATCGGTGGATT	54	427
		*gyr*B-R	CTGCCACTTGAGTTTGTACA		
*rrs*	Aminoglycosides (capreomycin)	*rrs*-F	AAACCTCTTTCACCATCGAC	59	1,329
		*rrs*-R	GTATCCATTGATGCTCGC		
*rpsL*	Streptomycin	rpsLfw	GGCCGACAAACAGAACGT	51	501
		rpsLrev	GTTCACCAACTGGGTGAC		

### Genotyping.

Spoligotyping (41, 42) was performed with 43 spacer probes coupled to MagPlex microspheres using the Luminex MagPix instrument (Luminex Corporation, Austin, TX, USA) and TB-SPOL Beamedex reagents (Beamedex SAS, Orsay, France). The spoligotyping PCR was carried out with 1× MasterMix (Applied Biological Materials Inc., Richmond, BC, Canada), containing the reaction buffer, 1 U *Taq* polymerase, 1.5 mM MgCl_2_, 0.2 mM dNTPs, with 0.6 μM each primer, and 5 μl of the Xpert sample remnant diluted 1:10 in water, in a 30-μl final volume. The amplification program was as follows: 95°C for 5 min; 45 cycles of 95°C for 1 min, 55°C for 1 min, and 72°C for 30 s; and a final extension of 72°C for 7 min. The spoligotyping patterns obtained were sent to the SITVIT Web database (http://www.pasteur-guadeloupe.fr:8081/SITVIT_ONLINE/description.jsp) for identification of the spoligotype international types.

### MIRU-VNTR typing.

Typing with four MIRU-VNTR systems (systems 1982, 3232, 3820, and 4120) reported to be hypervariable in strains of the Beijing lineage was performed with the primers described previously (18, 19) in a 50-μl final volume containing 1× PCR buffer, 1× Q-Solution, 500 nM dNTP, 1.5 mM MgCl_2_, 400 μM each primer, 1 U HotStarTaq DNA polymerase (Qiagen), and 5 μl of the Xpert sample remnant diluted 1:10 in water. The PCR parameters were as follows: 95°C for 15 min; 45 cycles of 94°C for 1 min, 59°C (MIRU 1982, 3820, 4102) or 60°C (MIRU 3232) for 1 min, and 72°C for 1.5 min; and a final extension of 72°C for 10 min. Eight microliters of each amplified PCR product was electrophoresed on a 1% agarose gel for 60 min at 160 V.

## Supplementary Material

Supplemental material
