# Supplementary material for: Use of GeneXpert Remnants for Drug Resistance Profiling and Molecular Epidemiology of Tuberculosis in Libreville, Gabon
Source: J Clin Microbiol. 2017 Jun 23;55(7):2105–15. doi: 10.1128/JCM.02257-16 (PMC5483912; doi:10.1128/JCM.02257-16)
Supplement: Supplemental material [file supp_55_7_2105__index.html]

Supplemental material 

# Use of GeneXpert Remnants for Drug Resistance Profiling and Molecular Epidemiology of Tuberculosis in Libreville, Gabon

## Supplemental material

- Supplemental file 1 -

  Fig. S1 (Amplification of hypervariable MIRU-VNTR loci from samples with Beijing and non-Beijing lineage spoligotypes)

  PDF, 765K
