# Supplementary material for: Use of GeneXpert Remnants for Drug Resistance Profiling and Molecular Epidemiology of Tuberculosis in Libreville, Gabon
Source: J Clin Microbiol. 2017 Jun 23;55(7):2105–15. doi: 10.1128/JCM.02257-16 (PMC5483912; doi:10.1128/JCM.02257-16)
Supplement: Supplemental material [file JCM.02257-16_zjm999095550s1.pdf]

Suppl. Figure 1.

Suppl. FIGURE 1.
Amplification of hypervariable MIRU-VNTR loci (1982, 3232, 3820, 4102) from samples with Beijing and non-Beijing lineage spoligotypes Lanes 1 to 5, Gabon samples with Beijing lineage spoligotype SIT1; Lane 7, Gabon sample with Beijing lineage spoligotype SIT260; lanes 6, 8, 10, 11 and 12, Gabon strains with spoligotypes not belonging to the Beijing lineage; Lane 9 was left intentionally empty; lanes A and B, strains from Paris Bichat-Claude Bernard Hospital with spoligotypes belonging to the Beijing lineage; lanes C and D, strains from Paris Bichat-Claude Bernard Hospital with spoligotypes not of the Beijing lineage

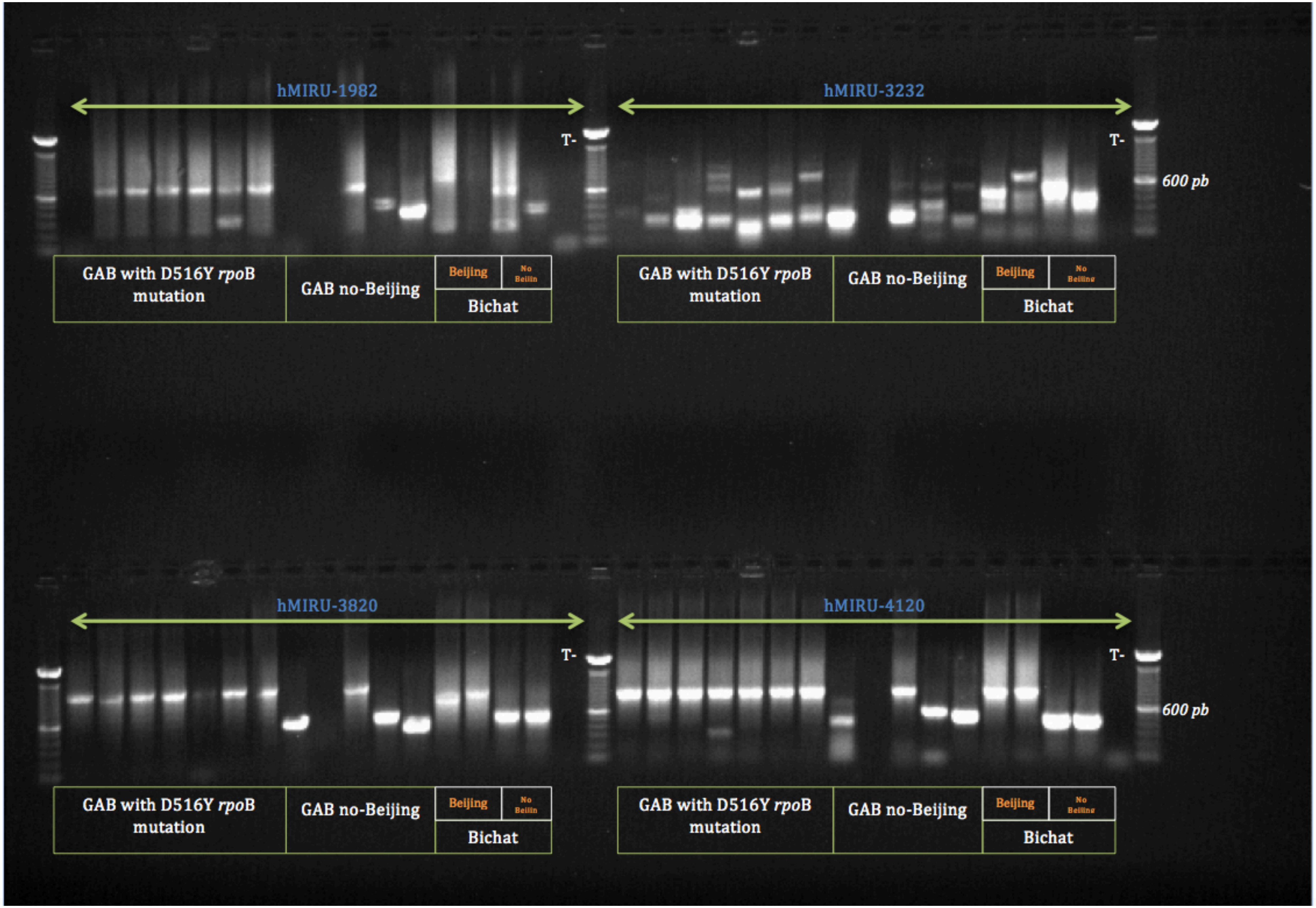
hMIRU-1982
T-
GAB with D516Y rpoB mutation
GAB no-Beijing
Beijing
Bichat
hMIRU-3232
T-
600 pb
GAB with D516Y rpoB mutation
GAB no-Beijing
Beijing
Bichat
hMIRU-3820
T-
GAB with D516Y rpoB mutation
GAB no-Beijing
Beijing
Bichat
hMIRU-4120
T-
600 pb
GAB with D516Y rpoB mutation
GAB no-Beijing
Beijing
Bichat
